# Evaluating the effectiveness of combined T4 and T3 therapy or desiccated thyroid versus T4 monotherapy in hypothyroidism: a systematic review and meta-analysis

**DOI:** 10.1186/s12902-024-01612-6

**Published:** 2024-06-14

**Authors:** Mahmoud Nassar, Ahmed Hassan, Shrouk Ramadan, Mariam Tarek Desouki, Malak A. Hassan, Ajay Chaudhuri

**Affiliations:** 1 Faculty of Medicine, Beni-Suef University, Beni-Suef, Egypt; 2 Jacobs School of Medicine and Biomedical Sciences, University of Buffalo, Buffalo, NY USA; 3 Department of Cardiology, Suez Medical Complex, Suez, Egypt; 4 Faculty of Medicine, Ain Shams University, Cairo, Egypt; 5 Faculty of Medicine, Alexandria University, Alexandria, Egypt

**Keywords:** Desiccated thyroid extract, Levothyroxine, Liothyronine, Hypothyroidism

## Abstract

**Background:**

Persistent symptoms in hypothyroid patients despite normalized TSH levels suggest the need for alternative treatments. This study aims to evaluate the effectiveness of combined T4 and T3 therapy or desiccated thyroid (DTE) compared to T4 monotherapy, with a focus on thyroid profile, lipid profile, and quality of life metrics.

**Methods:**

We conducted a systematic review in Embase, Medline/PubMed, and Web of Science up to 11/23/2023. We used the following keywords: “Armour Thyroid,” OR “Thyroid extract,” OR “Natural desiccated thyroid,” OR “Nature-Throid,” “desiccated thyroid,” OR “np thyroid,” OR “Synthroid,” OR “levothyroxine,” OR “Liothyronine,” “Cytomel,” OR “Thyroid USP,” OR “Unithroid.” AND “hypothyroidism. “ We only included RCTs and excluded non-RCT, case–control studies, and non-English articles.

**Results:**

From 6,394 identified records, 16 studies qualified after screening and eligibility checks. We included two studies on desiccated thyroid and 15 studies on combined therapy. In this meta-analysis, combination therapy with T4 + T3 revealed significantly lower Free T4 levels (mean difference (MD): -0.34; 95% CI: -0.47, -0.20), Total T4 levels (mean difference: -2.20; 95% CI: -3.03, -1.37), and GHQ-28 scores (MD: -2.89; 95% CI: -3.16, -2.63), compared to T4 monotherapy. Total T3 levels were significantly higher in combined therapy (MD: 29.82; 95% CI: 22.40, 37.25). The analyses demonstrated moderate to high heterogeneity. There was no significant difference in Heart Rate, SHBG, TSH, Lipid profile, TSQ-36, and BDI Score.

Subjects on DTE had significantly higher serum Total T3 levels (MD: 50.90; 95% CI: 42.39, 59.42) and significantly lower serum Total T4 (MD: -3.11; 95% CI: -3.64, -2.58) and Free T4 levels (MD: -0.50; 95% CI: -0.57, -0.43) compared to T4 monotherapy. Moreover, DTE treatment showed modestly higher TSH levels (MD: 0.49; 95% CI: 0.17, 0.80). The analyses indicated low heterogeneity. There was no significant difference in Heart Rate, SHBG, Lipid profile, TSQ-36, GHQ-28, and BDI Score.

**Conclusions:**

Our study revealed that combined therapy and DTE lead to higher T3 and lower T4 levels, compared to T4 monotherapy in hypothyroidism. However, no significant effects on heart rate, lipid profile, or quality of life were noted. Given the heterogeneity of results, personalized treatment approaches are recommended.

**Supplementary Information:**

The online version contains supplementary material available at 10.1186/s12902-024-01612-6.

## Introduction

Hypothyroidism is a prevalent endocrine disorder characterized by the thyroid gland’s inability to produce sufficient thyroid hormones, which are crucial for regulating metabolism and energy. This condition predominantly affects women and the elderly, impacting approximately 1–6% of the general population [1]. It can range in severity from mild, often asymptomatic forms to severe complications like myxedema, with symptoms including fatigue, weight gain, and cold intolerance [2, 3]. Despite the widespread use of synthetic levothyroxine (LT4) as standard treatment, which effectively normalizes thyroid-stimulating hormone (TSH) levels in most patients, a subset continues to experience persistent symptoms. This observation has spurred interest in exploring alternative treatment strategies, such as combined T4 and T3 therapy or desiccated thyroid extract (DTE) [4].

LT4 monotherapy has long been the cornerstone of hypothyroidism management, prized for its ability to standardize dosages and ensure consistent potency. While LT4 effectively alleviates the primary symptoms of hypothyroidism and prevents long-term health issues associated with thyroid hormone deficiency, approximately 5–10% of patients report persistent symptoms such as fatigue and mood disturbances, even when TSH levels are normalized [5, 6]. These persistent symptoms suggest that TSH levels may not fully reflect the thyroid hormone status at the tissue level, highlighting the potential inadequacies of LT4 monotherapy in some patients and underscoring the complexity of thyroid hormone metabolism, including genetic variations that affect hormone conversion [7].

Given the limitations of LT4 monotherapy, combined T4 and T3 therapy and DTE have been proposed as potential alternatives that could offer benefits over LT4 alone. Combined therapy, involving both LT4 and liothyronine (LT3), aims to more closely mimic the body’s natural thyroid hormone secretion patterns, potentially addressing the conversion issues from T4 to T3 seen in some patients [8]. Desiccated thyroid extract, made from dried and powdered animal thyroid glands, has been used for over a century and provides a natural source of both T4 and T3 hormones. Some patients prefer DTE due to its perceived effectiveness in controlling more persistent symptoms [7].

Investigating a broad spectrum of secondary outcome measures in the study of T4 monotherapy, DTE, and combined T4 + T3 therapies is vital for assessing their overall efficacy and safety. Such measures include thyroid and lipid profiles, which are crucial for understanding the metabolic impacts of each treatment on cardiovascular health [7], as well as quality of life indicators like the 36-point thyroid symptom questionnaire (TSQ-36), Beck Depression Inventory (BDI), and General Health Questionnaire (GHQ)-28 scores. These indicators provide essential insights into the subjective experience of symptoms and overall well-being, reflecting the treatments’ effectiveness from the patient’s perspective [9]. Additionally, incorporating a wide range of outcomes facilitates a comprehensive evaluation of the multifaceted impacts of thyroid treatments, enabling more tailored and effective patient care [10]. This holistic approach ensures that all potential benefits and drawbacks of the therapies are thoroughly assessed, aiding in personalized patient management decisions.

This systematic review and meta-analysis aim to rigorously evaluate the effectiveness of combined T4 and T3 therapy, or DTE, compared to T4 monotherapy. The focus is on comprehensive endpoints including thyroid profile, lipid profile, and quality of life metrics such as the TSQ-36, BDI scores, and GHQ-28 scores. By incorporating randomized clinical studies, this study seeks to provide a clearer understanding of the impact of these alternative therapies on clinical and laboratory measurements, potentially guiding more personalized and effective treatment strategies for hypothyroidism.

## Methods

The methodology began with a strategic literature search employing a Boolean approach across several medical databases, including Embase, Medline/PubMed, and Web of Science. We scanned these databases from their inception up to November 23, 2023. The search was guided by a set of carefully chosen keywords encompassing various nomenclatures for thyroid medications and treatments, such as “Armour Thyroid,” OR “Natural desiccated thyroid,” OR “Thyroid extract,” OR “Desiccated Thyroid,” OR “Thyroid USP,” OR “Unithroid,” OR “Synthroid,” OR “Levothyroxine,” OR “Liothyronine,” OR “Cytomel,” AND “hypothyroidism” (Supplementary Table 1).

We included human studies, ages 18 years or more, who had been diagnosed with primary hypothyroidism. Our inclusion criteria were strictly limited to randomized controlled trials (RCTs) to ensure the highest level of evidence. We included RTC that reported combined T4/T3 or DTE and levothyroxine as the intended therapeutic intervention and control, respectively, for patients with hypothyroidism. We excluded retrospective studies, review articles, abstracts, case–control studies, and articles not published in English. We excluded studies that included pregnancy and pediatric patients.

The initial search yielded a substantial number of records exported to the Covidance website, from which duplicates were removed. The remaining records underwent a screening process, first by title and abstract, followed by a full-text review to assess eligibility. Two independent coauthors did the screening process. This systematic approach ensured that only the most relevant and high-quality studies were included in our analysis. The data was extracted to an Excel sheet and double-checked by two independent coauthors. Subsequently, the measurement units were standardized to incorporate them into the pooled analysis.

The final selection of studies incorporated into our meta-analysis comprised a balanced mix of research focusing on desiccated thyroid and combined therapy. We extracted from these studies and were then meticulously analyzed. We calculated mean differences (MDs) with 95% confidence intervals (CIs) for various outcomes, such as levels of Free T4, Total T4, Total T3, and scores on GHQ-28, TSQ-36, and BDI. Additionally, we evaluated the heterogeneity of the studies to understand the variability in their results.

Through this methodical approach, our study aimed to provide a comprehensive and evidence-based evaluation of the effectiveness of combined T4 and T3 therapy or desiccated thyroid in managing hypothyroidism compared to conventional T4 monotherapy. The review protocol was registered in PROSPERO in advance: CRD42023486957.

### Data extraction

The inclusion criteria were assessed, and data extraction was performed through an independent review of titles, abstracts, and studies by four investigators (A.H, M.D, S.R, and MH). Any disagreements were resolved through discussion and agreement with MN. The data extracted for qualitative synthesis included center, recruitment of patients, year of study, study design, sample size, and population age (in years).

### Risk-of-bias assessment

We utilized the updated Cochrane risk-of-bias tool for randomized trials (RoB 2) [11] to assess the potential for bias in the included clinical trials. This evaluation included an assessment of the randomization process, concealment of the allocation sequence, deviations from the intended interventions, utilization of appropriate analysis to estimate the effect of assignment to intervention, measurement of the outcome, selection of the reported results, and overall risk of bias. The assessment of the methodological quality of the studies was classified as either low risk, with some concerns, or high risk of bias.

### Study outcomes

Our endpoints of the current investigation included thyroid profile, lipid profile, heart rate, sex hormone-binding globulin (SHBG), quality of life metrics, including the 36-point thyroid symptom questionnaire (TSQ-36), BDI Scores, and GHQ-28 scores.

### Statistical analysis

The statistical analysis was conducted using Review Manager (RevMan) software. Continuous variables were analyzed using mean differences (MDs) or standardized mean differences (SMDs) with 95% confidence intervals (CIs), depending on the uniformity of measurement scales across studies. Forest plots were used to represent the individual and pooled results of the studies visually.

Heterogeneity among studies was quantified using the *I*^2^ statistic, which measures the percentage of total variation across studies due to heterogeneity rather than chance. An *I*^2^ value greater than 50% indicated substantial heterogeneity, prompting further subgroup or sensitivity analyses to explore potential sources of this variation.

## Results

**Fig. 1:**
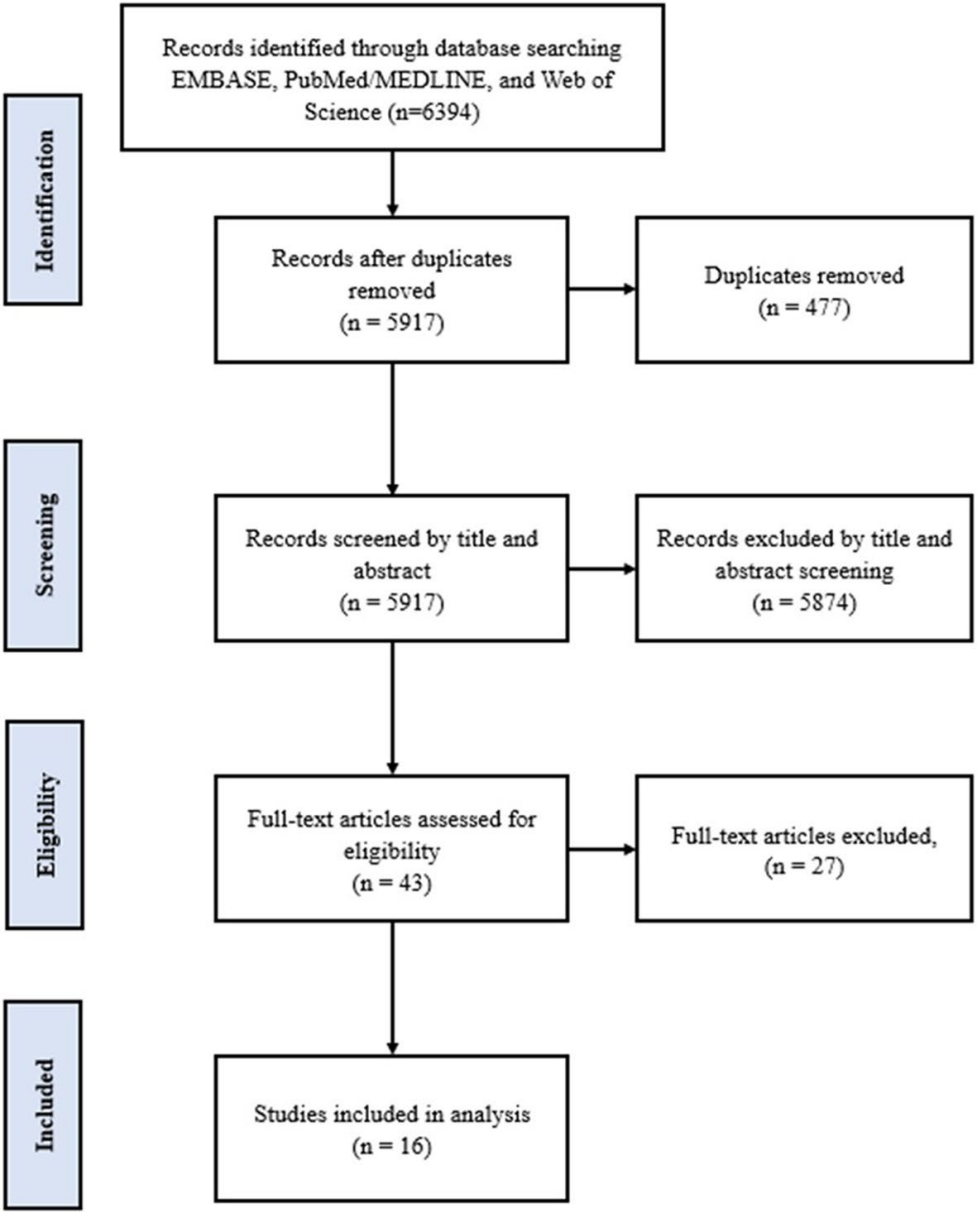
PRISMA Flow diagram of literature search and study selection process

A thorough search of the EMBASE, PubMed/MEDLINE, and Web of Science databases yielded 6,394 records. Out of these, 477 duplicate records were detected and eliminated, resulting in a remaining count of 5,917 records to be examined. By reviewing titles and abstracts, we excluded 5,874 records that did not meet our predefined eligibility criteria during the screening process. A meticulous evaluation was conducted to determine the eligibility of the remaining 43 full-text articles. Out of these, 27 articles were excluded. In the end, 16 studies were considered suitable and included in the analysis (Supplementary Tables 2 and 3) (Fig. 1).

### Effect of T4 + T3 on thyroid hormone levels

**Fig. 2:**
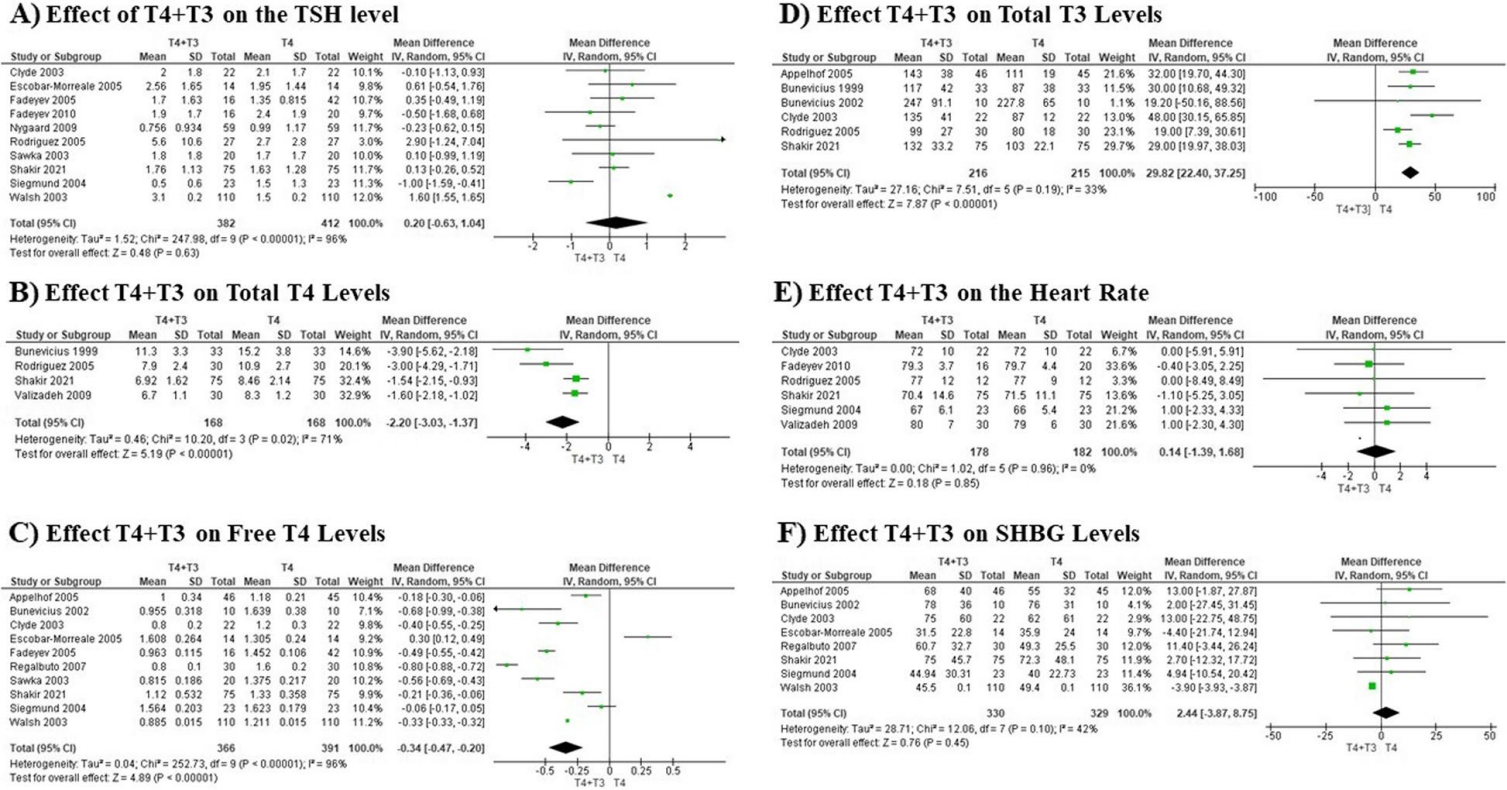
Forest plots illustrating the effects of combined T4 + T3 therapy vs. T4 monotherapy on TSH, Total T4, Free T4, Total T3, Heart rate, and SHBG levels

Our meta-analysis explored the effects of combined T4 + T3 therapy versus T4 monotherapy on thyroid hormone levels in patients with hypothyroidism, drawing from studies with diverse methodologies and participant demographics. The analysis of TSH levels in 794 participants across ten studies [9, 12–20], showed no significant difference with a mean change of 0.20 (95% CI: -0.63 to 1.04), confirmed by statistical tests indicating non-significance (*P* = 0.63) (Fig. 2A). In contrast, the examination of total T4 levels from four studies [9, 17, 21, 22], with 336 participants revealed that subjects on combined therapy had significantly lower levels (mean difference: -2.20, 95% CI: -3.03 to -1.37), with results proving statistically significant (*P* < 0.00001) (Fig. 2B). Similarly, analysis of free T4 levels from 757 participants across 10 studies demonstrated a significantly lower level (mean difference: -0.34, 95% CI: -0.47 to -0.20), also statistically significant (*P* < 0.00001) (Fig. 2C). Furthermore, the investigation of total T3 levels in 431 participants from six studies found a significantly higher level with combined therapy (mean difference: 29.82, 95% CI: 22.40 to 37.25), with this effect confirmed as statistically significant (*P* < 0.00001) (Fig. 2D). The studies demonstrated varying degrees of heterogeneity. These results highlight the varying impacts of combined T4 + T3 therapy on thyroid hormones, indicating significant differences from traditional T4 monotherapy in the treatment of hypothyroidism (Table 1).

**Table 1:** Summary of meta-analysis results comparing combined T4 + T3 therapy to T4 monotherapy across various clinical outcomes

Outcome	No. of RCT	Total number of patients		Heterogeneity				Test for overall effect		Mean Difference	95% CI
		**T4 + T3**	**T4**	**Tau**^**2**^	**Chi**^**2**^	**df**	***I***^**2**^	**Z**	***P***		
TSH	10	382	412	1.52	247.98	9 (*P* < 0.00001)	96%	0.48	0.63	0.20	[-0.63, 1.04]
Total T4	4	168	168	0.46	10.20	3 (*P* = 0.02)	71%	5.19	< 0.00001	-2.20	[-3.03, -1.37]
Free T4	10	366	391	0.04	252.73	9 (*P* < 00001)	96%	4.89	< 0.00001	-0.34	[-0.47, -0.20]
Total T3	6	216	215	27.16	7.51	5 (*P* = 0.19)	33%	7.87	< 0.00001	29.82	[22.40, 37.25]
Heart rate	6	178	182	0.00	1.02	5 (*P* = 0.96)	0%	0.18	0.85	0.14	[-1.39, 1.68]
SHBG	8	330	329	28.71	12.06	7 (*P* = 0.10)	42%	0.76	0.45	2.44	[-3.87, 8.75]
LDL	6	180	184	45.72	7.47	5 (*P* = 0.19)	33%	1.18	0.24	-5.79	[-15.39, 3.81]
Total cholesterol	10	414	418	90.48	26.88	8 (*P* = 0.0007)	70%	1.29	0.20	-5.47	[-13.80, 2.85]
HDL	4	150	150	18.33	9.29	3 (*P* = 0.03)	68%	0.84	0.40	2.30	[-3.09, 7.69]
Triglyceride	7	259	258	157.24	10.64	6 (*P* = 0.10)	44%	0.44	0.66	-3.23	[-17.72, 11.27]
TSQ-36	2	185	185	0.95	2.41	1 (*P* = 0.12)	58%	0.33	0.74	0.27	[-1.34, 1.88]
GHQ-28	2	140	140	4.64	2.57	1 (*P* = 0.11)	61%	0.95	0.34	1.71	[-1.82, 5.23]
BDI score	2	85	85	0.00	0.82	1 (*P* = 0.37)	0%	0.38	0.70	-0.39	[-2.41, 1.62]

*RCT* Randomized Controlled Trials, *T4* Thyroxine, *T3* Triiodothyronine, *Tau*^*2*^ Tau-squared, *Chi*^2^ Chi-squared, *df* Degrees of Freedom, *I*^*2*^ I-squared (Heterogeneity), *Z* Z-score, *P* P-value, *SHBG* Sex Hormone-Binding Globulin, *LDL* Low-Density Lipoprotein, *HDL* High-Density Lipoprotein, *TSQ-36* Thyroid Symptom Questionnaire-36, *GHQ-28* General Health Questionnaire-28, *BDI* Beck Depression Inventory

### Effect of T4 + T3 on heart rate and SHBG levels

Our meta-analysis assessed the effects of combining T4 with T3 compared to T4 monotherapy on heart rate and serum sex hormone-binding globulin (SHBG) levels in hypothyroid patients. The study involving 360 participants found no significant difference in heart rate between the two therapies, with consistent results across all studies indicating stable heart rate outcomes regardless of the treatment (Fig. 2E). Similarly, an analysis of SHBG levels from 659 participants showed no significant impact from the combined therapy, despite moderate variability among the studies. Overall, both outcomes suggest that adding T3 to T4 therapy does not significantly affect heart rate or SHBG levels compared to T4 alone (Fig. 2F) (Table 1).

### Effect of T4 + T3 on lipid profile

**Fig. 3:**
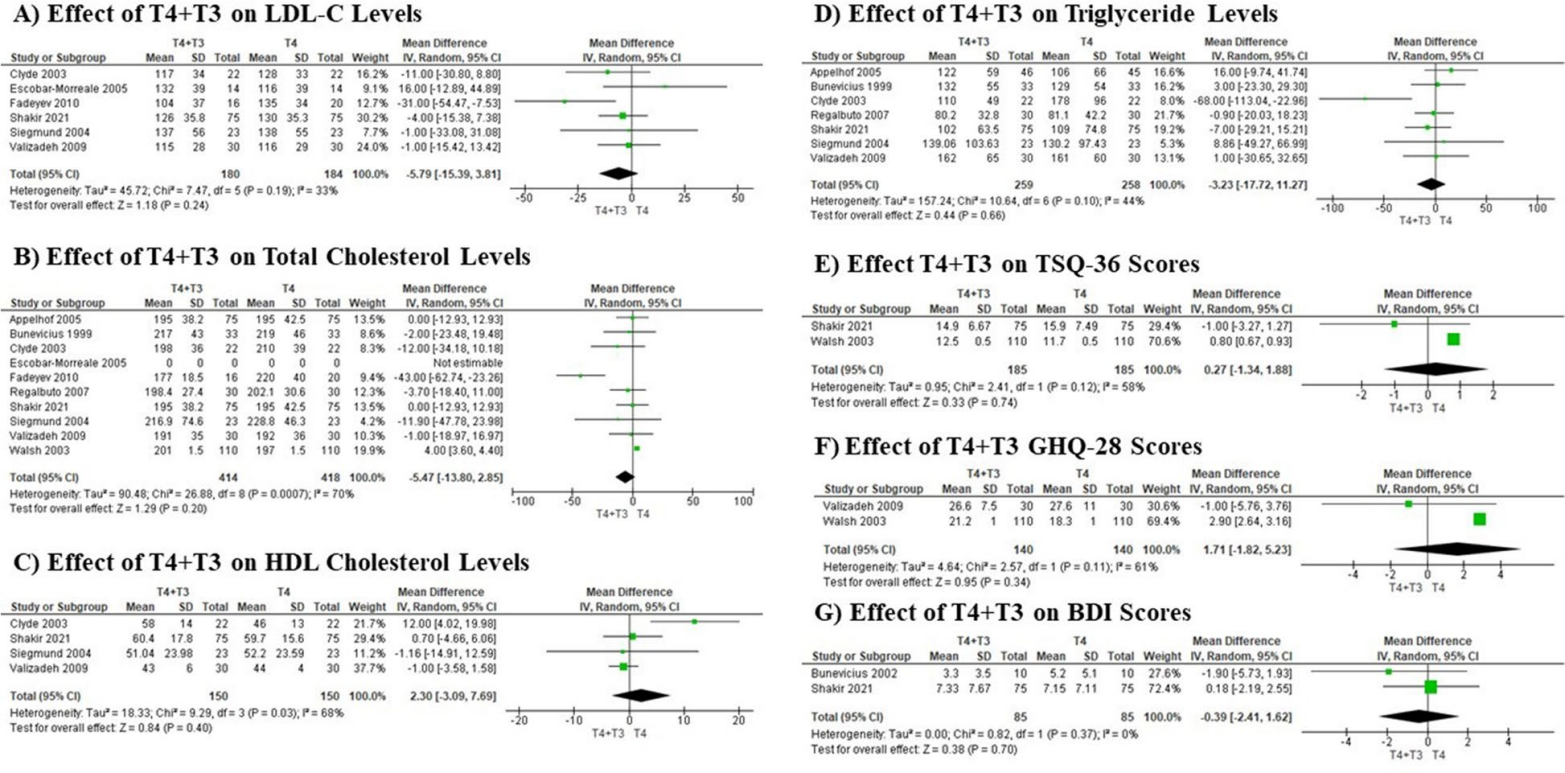
Forest plots illustrating the effects of combined T4 + T3 Therapy vs. T4 Monotherapy on lipid profiles, quality of life, and mental health outcomes

Our meta-analysis evaluated the effects of combined T4 + T3 therapy versus T4 monotherapy on lipid profiles in patients with hypothyroidism, specifically examining LDL-C, total cholesterol, HDL cholesterol, and triglyceride levels. For LDL-C, data from 364 participants across six studies indicated a non-significant trend towards decreased levels with combined therapy, though the result was not statistically significant (Fig. 3A). Similarly, the analysis of total cholesterol levels in 832 participants from nine studies showed no significant differences between the treatment groups (Fig. 3B). The assessment of HDL cholesterol from four studies with 300 participants also revealed no significant change, with high heterogeneity indicating inconsistent effects across different studies (Fig. 3C). Lastly, the evaluation of triglyceride levels from seven studies and 517 participants showed no substantial differences, with low heterogeneity pointing to a generally consistent lack of effect across studies (Fig. 3D). Overall, these results suggest that adding T3 to T4 therapy does not significantly alter lipid levels compared to T4 monotherapy in hypothyroid patients (Table 1).

### Effect of T4 + T3 on psychological and quality-of-life measures

Our meta-analysis assessed the effects of combined T4 + T3 therapy versus T4 monotherapy on psychological and quality-of-life measures across different studies, examining TSQ-36, GHQ-28, and BDI scores. The analysis showed no significant impact on quality of life, as measured by TSQ-36 scores, with a mean difference of 0.27 (95% CI: -1.34 to 1.88) among 370 participants, suggesting that combined therapy does not significantly enhance quality of life compared to monotherapy (Fig. 3E). Additionally, the analysis showed no significant impact in mental health and well-being with combined therapy, as GHQ-28 scores among 280 participants revealed score (mean difference: 1.71, 95% CI: -1.82 to 5.23), with a highly significant effect and no heterogeneity, pointing to a reliable benefit in mental health (Fig. 3F). However, depressive symptoms measured by BDI scores in 170 participants showed no significant changes (mean difference: -0.39, 95% CI: -2.41 to 1.62), confirming that combined T4 + T3 therapy does not significantly affect depressive symptoms (Fig. 3G). These findings suggest that while combined T4 + T3 therapy significantly improves mental health, it does not have a marked impact on overall quality of life or depressive symptoms compared to T4 monotherapy (Table 1).

The risk of bias within the included studies was systematically evaluated across seven key domains, as shown in the Risk of Bias Summary (Suppl. Figure 1). Most studies exhibited a low risk of bias in random sequence generation and allocation concealment, suggesting a strong methodological framework for randomization in these trials. However, there were instances of high risk of bias in domains such as blinding of participants and personnel, and incomplete outcome data, indicating areas where the study outcomes could be influenced by knowledge of the intervention or by missing data. The Risk of Bias Graph (Suppl. Figure 2) illustrates the proportion of studies with low, unclear, and high risk of bias within each domain. Notably, while most studies maintained a low risk of bias in random sequence generation, concerns were more prevalent regarding performance and detection bias, where blinding may not have been adequately implemented or was not possible due to the nature of the intervention. These figures collectively indicate that while the body of evidence is generally robust, certain biases should be taken into consideration when interpreting the results of the meta-analysis.”

### Effect of DTE on thyroid hormone levels

**Fig. 4:**
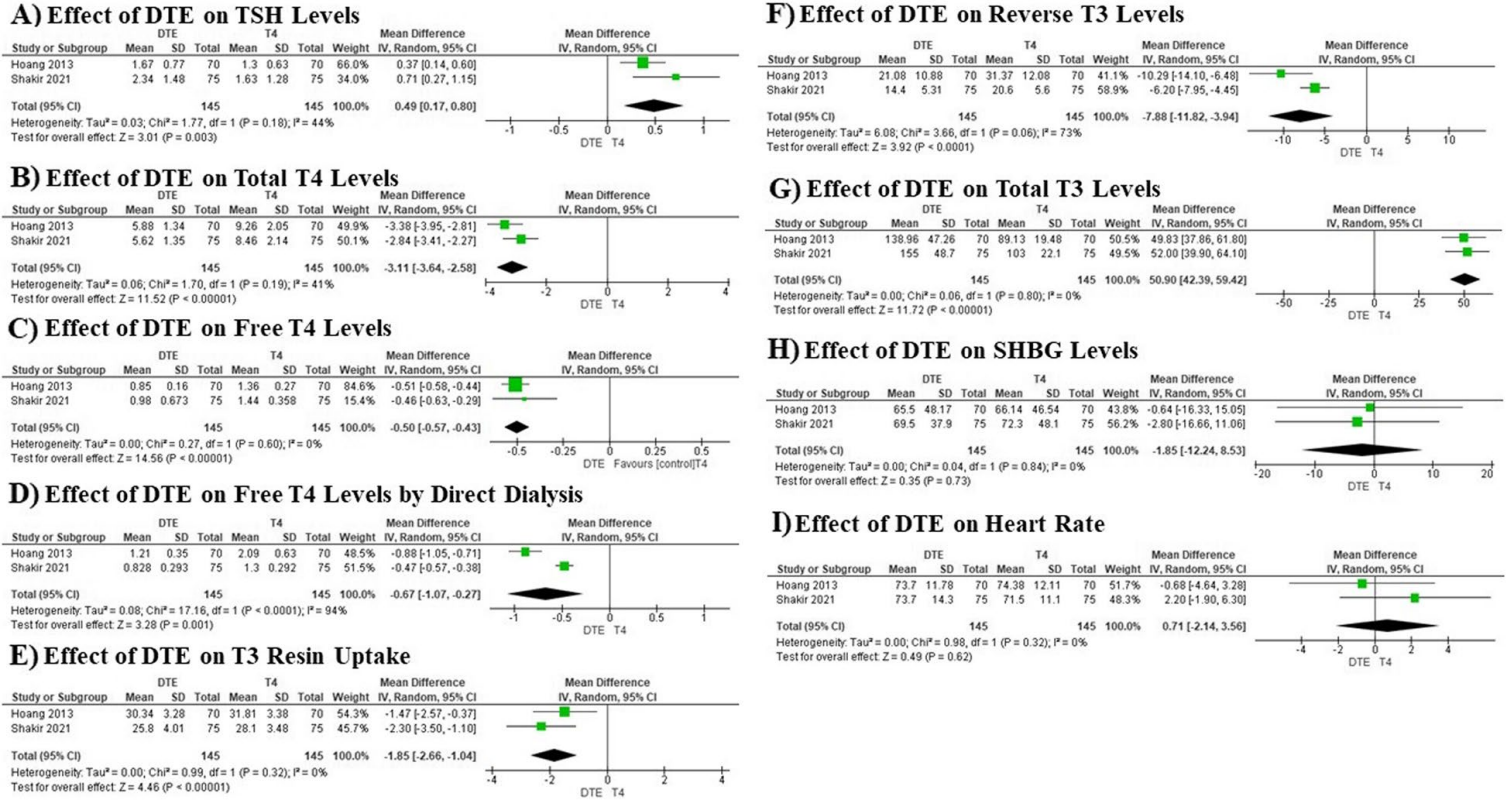
Forest plots illustrating the effects of DTE therapy vs. T4 monotherapy on lipid profiles, quality of life, and mental health outcomes

Our meta-analysis evaluated the effects of DTE versus T4 monotherapy on a variety of thyroid-related parameters across several studies, involving a total of 290 participants. The analysis revealed that DTE treatment led to statistically significant higher TSH levels with a mean difference of 0.49, suggesting higher TSH levels compared to T4 monotherapy (Fig. 4A). Similarly, there were significantly lower total T4 levels with a mean difference of -3.11, indicating a robust low level due to DTE treatment (Fig. 4B). For free T4, both methods—standard measurement and direct dialysis—showed significantly lower levels with mean differences of -0.50 and -0.67, respectively (Fig. 4C and 4D). Furthermore, the meta-analysis noted a significantly low level of T3 resin uptake with a mean difference of -1.85, demonstrating a consistent effect across studies (Fig. 4E). In terms of reverse T3 levels, the treatment with DTE also resulted in a significant lowering, with a mean difference of -7.88 (Fig. 4F). Most notably, there were significantly higher total T3 levels, with a mean difference of 50.90, suggesting a substantial elevation in patients treated with DTE as compared to those on T4 monotherapy (Fig. 4G). This overall analysis highlights the considerable impact of DTE on thyroid function and hormone levels, indicating distinct effects in various thyroid-related parameters when compared to standard T4 monotherapy (Table 2).

**Table 2:** Summary of meta-analysis results comparing DTE therapy to T4 monotherapy across various clinical outcomes

Outcome	No. of RCT	Total number of patients		Heterogeneity				Test for overall effect		Mean Difference	95% CI
		**DTE**	**T4**	**Tau**^**2**^	**Chi**^**2**^	**df**	***I***^***2***^	**Z**	***P***		
TSH	2	145	145	0.03	1.77	1 (*P* = 0.18)	44%	3.01	0.003	0.49	[0.17, 0.80]
Total T4	2	145	145	0.06	1.70	1 (*P* = 0.19)	41%	11.52	< 0.00001	-3.11	[-3.64, -2.58]
Free T4	2	145	145	0.00	0.27	1 (*P* = 0.60)	0%	14.56	< 0.00001	-0.50	[-0.57, -0.43]
Free T4 by direct dialysis	2	145	145	0.08	17.16	1 (*P* < 0.0001)	94%	3.28	0.001	-0.67	[-1.07, -0.27]
T3 Resin uptake	2	145	145	0.00	0.99	1 (*P* = 0.32)	0%	4.46	< 0.00001	-1.85	[-2.66, -1.04]
Reverse T3	2	145	145	6.08	3.66	1 (*P* = 0.06)	73%	3.92	< 0.0001	-7.88	[-11.82, -3.94]
Total T3	2	145	145	0.00	0.06	1 (*P* = 0.80)	0%	11.72	< 0.00001	50.90	[42.39, 59.42]
Total cholesterol	2	145	145	0.00	0.44	1 (*P* = 0.51)	0%	0.50	0.62	-2.19	[-10.76, 6.39]
LDL	2	145	145	0.00	0.01	1 (*P* = 0.94)	0%	0.71	0.48	-2.66	[-10.02, 4.70]
HDL	2	145	145	0.00	0.64	1 (*P* = 0.42)	0%	0.46	0.65	-0.87	[-4.60, 2.86]
Triglyceride	2	145	145	0.00	0.03	1 (*P* = 0.85)	0%	0.22	0.82	-1.65	[-16.03, 12.73]
SHBG	2	145	145	0.00	0.04	1 (*P* = 0.84)	0%	0.35	0.73	-1.85	[-12.24. 8.53]
Heart rate	2	145	145	0.00	0.98	1 (*P* = 0.32)	0%	0.49	0.62	0.71	[-2.14, 3.56]
GHQ-12	2	145	145	0.00	0.67	1 (*P* = 0.41)	0%	1.30	0.19	-0.78	[-1.95, 0.39]
TSQ-36	2	145	145	0.00	0.00	1 (*P* = 1.00)	0%	1.65	0.10	-1.40	[-3.06, 0.26]
BDI score	2	145	145	0.00	0.05	1 (*P* = 0.82)	0%	0.44	0.66	-0.30	[-1.65, 1.04]
AMI score	2	145	145	0.00	0.47	1 (*P* = 0.49)	0%	0.69	0.49	1.09	[-2.01, 4.19]
VMI score	2	145	145	0.00	0.18	1 (*P* = 0.67)	0%	0.16	0.87	-0.23	[-3.04, 2.58]
VWMI score	2	145	145	0.00	0.17	1 (*P* = 0.68)	0%	0.12	0.91	-0.22	[-3.90, 3.47]
IMI score	2	145	145	0.00	0.88	1 (*P* = 0.35)	0%	0.27	0.79	-0.44	[-3.58, 2.71]
DMI score	2	145	145	0.00	0.32	1 (*P* = 0.57)	0%	0.42	0.68	0.62	[-2.31, 3.56]

*RCT* Randomized Controlled Trials, *DTE* Desiccated Thyroid Extract, *T4* Thyroxine, *Tau*^*2*^ Tau-squared, *Chi*^*2*^ Chi-squared, *df* Degrees of Freedom, *I*^*2*^ I-squared (Heterogeneity), *Z* Z-score, *P* P-value, *TSH* Thyroid-Stimulating Hormone, *SHBG* Sex Hormone-Binding Globulin, *LDL* Low-Density Lipoprotein, *HDL* High-Density Lipoprotein, *GHQ-12* General Health Questionnaire-12, *TSQ-36* Thyroid Symptom Questionnaire-36, *BDI* Beck Depression Inventory, *AMI* Acute Myocardial Infarction Score, *VMI* Visual Memory Index, *VWMI* Verbal Working Memory Index, *IMI* Integrative Mental Index, *DMI* Deep Mental Index

### Effect of DTE on heart rate and SHBG levels

In our meta-analysis, we investigated the effects of DTE versus T4 monotherapy on SHBG levels and heart rate in patients undergoing treatment for thyroid conditions, drawing from two studies each with 290 participants. For SHBG levels, the analysis showed a mean difference of -1.85 (95% CI: -12.24 to 8.53), indicating no significant difference between the DTE and T4 monotherapy groups. The heterogeneity was extremely low (*I*^2^ = 0%), and the Chi^2^ statistic was 0.04, underscoring a high consistency across the studies. A Z-test confirmed this finding with a P-value of 0.73, further substantiating that the changes in SHBG levels are not statistically significant when comparing the two treatments (Fig. 4H). Similarly, the effect of DTE on heart rate also revealed no significant differences, with a mean difference of 0.71 (95% CI: -2.14 to 3.56). The heterogeneity among the studies was again very low (*I*^2^ = 0%), suggesting consistent results across the data. The P-value from the Z-test was 0.62, reinforcing that DTE does not significantly affect heart rate compared to T4 monotherapy (Fig. 4I) (Table 2).

### Effect of DTE on lipid profile

**Fig. 5:**
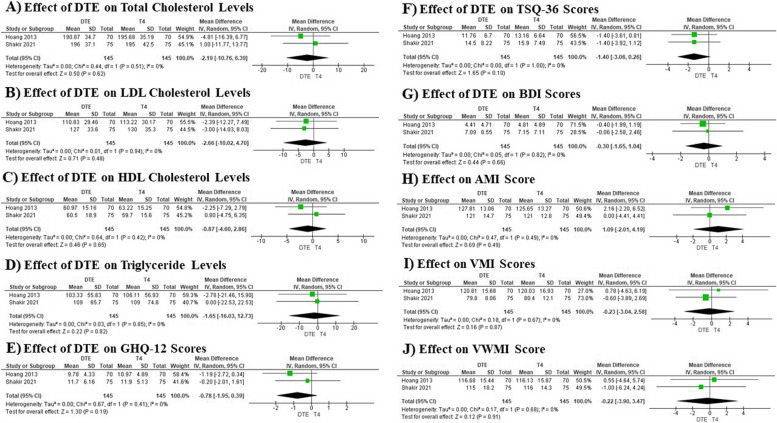
Forest plots illustrating the effects of DTE therapy vs. T4 monotherapy on lipid profiles, quality of life, and mental health outcomes

Our meta-analysis examined the impact of DTE compared to T4 monotherapy on cholesterol and triglyceride levels across two studies involving 290 participants each. The findings indicated no statistically significant differences in the lipid profiles between the two treatment groups. Specifically, the analysis of total cholesterol levels showed a mean difference of -2.19 (95% CI: -10.76 to 6.39), with negligible heterogeneity (*I*^2^ = 0%), and a Z-test P-value of 0.62, confirming the lack of significant difference (Fig. 5A). Similarly, LDL cholesterol levels presented a mean difference of -2.66 (95% CI: -10.02 to 4.70), also demonstrating consistency across studies (*I*^2^ = 0%) and a non-significant Z-test result (*P* = 0.48) (Fig. 5B). For HDL cholesterol, the results showed a mean difference of -0.87 (95% CI: -4.60 to 2.86), with no heterogeneity among the studies (*I*^2^ = 0%) and a Z-test P-value of 0.65, indicating no substantial change (Fig. 5C). Lastly, the analysis of triglyceride levels revealed a mean difference of -1.65 (95% CI: -16.03 to 12.73), with a similarly low heterogeneity (*I*^2^ = 0%) and a Z-test P-value of 0.82, underscoring the non-significant effect (Fig. 5D) (Table 2).

**Fig. 6:**
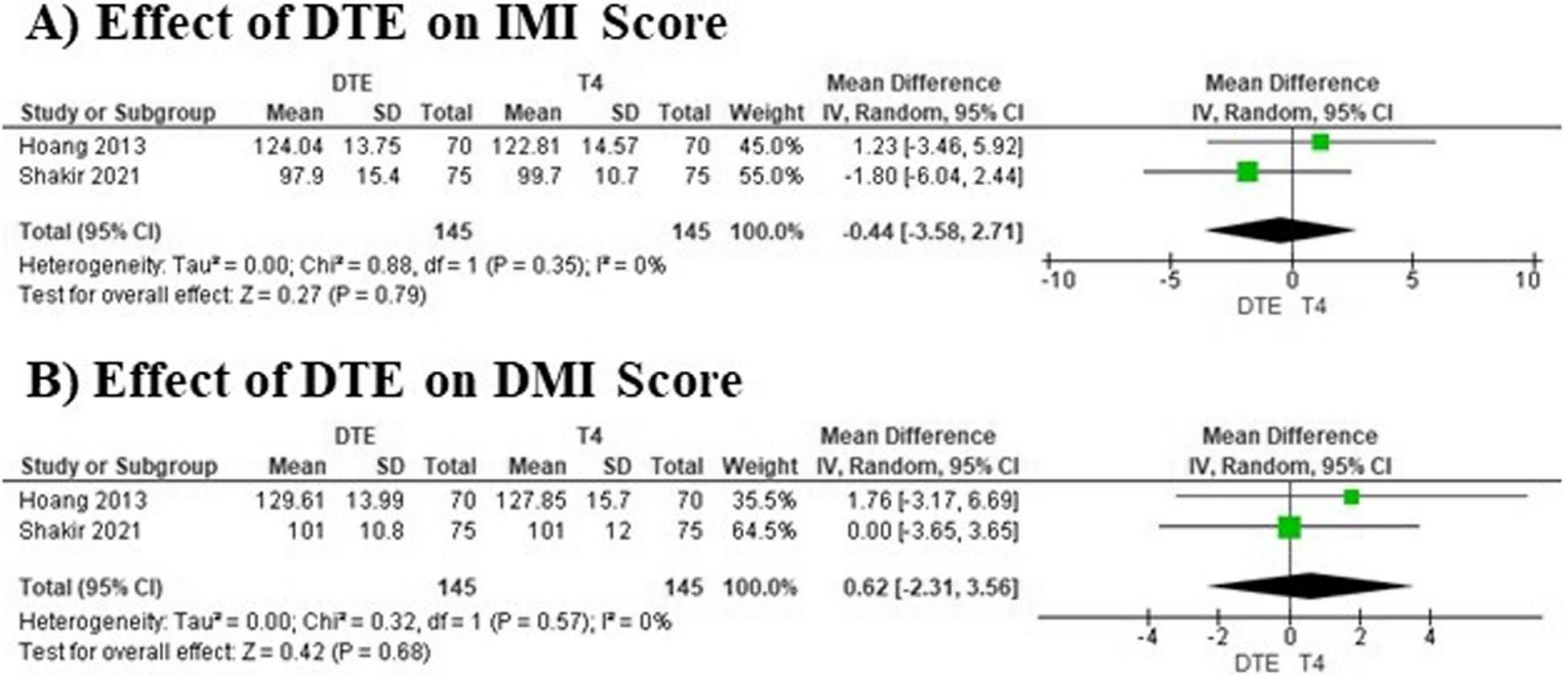
Forest plots illustrating the effects of DTE therapy vs. T4 monotherapy on IMI and DMI

Our comprehensive meta-analysis explored the effects of DTE vs. T4 monotherapy on various psychological and quality-of-life metrics, utilizing data from two studies with a total of 290 participants for each measure. The analysis examined GHQ-12 scores, indicating mental well-being, and found no notable difference between the two therapies with a mean difference of -0.78, suggesting similar effects on mental health (*I*^2^ = 0%) (Fig. 5E). Similarly, TSQ-36 scores, which assess the quality-of-life symptoms, showed a non-significant trend towards improvement with DTE, though not reaching statistical significance (mean difference: -1.40) (Fig. 5F). Depressive symptoms, measured by BDI scores, also displayed no significant differences between treatments (mean difference: -0.30), with consistent findings across individual studies (Fig. 5G). Likewise, AMI scores related to mental agility, VMI scores for visual memory, and VWMI scores for verbal working memory all demonstrated no significant differences between DTE and T4 monotherapy, indicating stable cognitive function across treatments. The mean differences for AMI, VMI, and VWMI were 1.09, -0.23, and -0.22 respectively, all with minimal heterogeneity (*I*^2^ = 0%) (Fig. 5H-J). Additionally, IMI and DMI scores, which may reflect integrative and deeper cognitive processes, showed no significant impacts from either treatment, with mean differences of -0.44 and 0.62 respectively. These findings were also characterized by a lack of heterogeneity (Fig. 6A, 6B) (Table 2).

The risk of bias within the included studies was systematically evaluated across seven key domains, as shown in the Risk of Bias Summary (Suppl. Figure 3 and 4). All studies exhibited a low risk of bias in random sequence generation and allocation concealment, suggesting a strong methodological framework for randomization in these trials.

## Discussion

Our meta-analysis provides comprehensive insights into the effects of thyroid hormone replacement therapies. The results indicate that combined T4 + T3 therapy does not significantly alter TSH levels compared to T4 monotherapy. This finding is crucial for clinicians, as TSH is a primary target for management of hypothyroidism. The high heterogeneity observed might reflect methodological differences across studies, warranting cautious interpretation. Most of the included studies are insignificant [9, 12–18] except for two studies, one showed a lower TSH, and the other showed a higher TSH [19, 20]. In contrast, total T4 and free T4 levels are significantly lower while total T3 levels are higher with combined therapy. This is consistent with the pharmacological profiles of the therapies as T3 addition would lead to lower T4 requirements and levels. Despite lower T4 levels, TSH is being maintained as it is primarily controlled by T3 concentrations within the pituitary. In comparing DTE with T4 monotherapy, our analysis revealed significantly higher (but still within normal range) TSH and total T3 levels with DTE, accompanied by decreases in total and free T4 levels. These changes reflect the distinctive pharmacokinetic profiles of DTE.

The analysis showed no significant differences in heart rate, LDL-C, total cholesterol, and HDL cholesterol levels between T4 + T3 therapy and T4 monotherapy and between DTE and T4 monotherapy groups. These results suggest that choosing between combined therapy and monotherapy may not have a big effect on the cardiovascular risk profiles of hypothyroid patients. This is important to consider because thyroid dysfunction is known to affect cardiovascular health negatively.

Our findings also indicate no significant impact of combined therapy on serum SHBG, triglyceride levels, or quality of life measures (TSQ-36 scores). However, a notable improvement in mental health and well-being was observed, as indicated by lower GHQ-28 scores with combined therapy. This suggests the potential benefits of combined therapy in the psychological aspect of hypothyroidism management.

The GHQ-12 scores and other mental health parameters (BDI, AMI, VMI, VWMI, IMI, and DMI) showed no significant differences between the DTE and T4 monotherapy groups, suggesting similar mental health outcomes with both treatments.

Our results reveal that combined T4 + T3 therapy does not significantly alter TSH levels, yet it influences free T3 levels, aligning with findings from previous research [23]. Our analysis also shows a notably lower total T4 and a higher total T3 with combined therapy. This supports that combined therapy improves T3 levels, which could help patients with issues in the conversion of T4 to T3 in the peripheral tissues. [23]. The variable impacts on metabolic parameters and quality of life, as noted in prior studies on T4 + T3 therapy, may be attributed to the subjective nature of quality-of-life assessments and differing metrics utilized across studies [24]. Furthermore, our findings show that TSH and T3 levels change significantly with DTE. This suggests that natural formulations, which contain both T3 and T4, may have different biochemical but not clinical effects when compared to synthetic therapies. These observed discrepancies could stem from heterogeneity in patient populations, study designs, dosing regimens, and the sensitivity of assays used to measure thyroid hormone levels, highlighting the complexity and individual variability in thyroid hormone replacement therapy responses.

Thyroxine (T4) has a half-life of approximately 7 days, serving as a stable, long-acting hormone reservoir for conversion to the more active triiodothyronine (T3), which has a shorter half-life of about 1 day, reflecting its potent and rapid-acting metabolic effects on energy expenditure and thermogenesis; meanwhile, Thyroid-Stimulating Hormone (TSH) has a half-life of about 60–80 min, allowing for rapid adjustments in response to the body’s thyroid hormone needs [25–27].

The potential mechanisms underlying the observed effects of combined T4 and T3 therapy, as compared to levothyroxine (T4) alone, involve several complex factors that vary among individuals. This combined therapy aims to more closely mimic the natural secretion of the thyroid gland, potentially restoring physiological levels of T3 and T4 across various tissues [28]. T3, the more active hormone, may enhance metabolic effects in peripheral tissues more directly than T4, a prohormone, thus ameliorating hypothyroid symptoms [29]. Genetic variations in thyroid hormone transport and metabolism, such as those involving deiodinase enzymes, might make certain individuals more responsive to T3 supplementation, particularly those with specific genetic polymorphisms affecting the conversion of T4 to T3 [30]. Despite mixed results in clinical trials, patient preference for combined therapy over T4 alone often arises from subjective improvements in symptoms like fatigue and cognitive function, which are not consistently quantifiable by standard clinical parameters [31]. While the evidence remains mixed, and most professional guidelines still recommend T4 monotherapy as the standard treatment, combined therapy may offer benefits to specific patients, particularly those who do not efficiently convert T4 to T3.

DTE, commonly known as Nature Thyroid, presents a distinct biochemical profile compared to synthetic levothyroxine (LT4), containing not only T4 and T3 but also additional thyroid hormones like T2 and calcitonin. This comprehensive hormonal composition may more closely mimic the natural output of the thyroid gland, potentially leading to improved symptom management for some patients [32]. Many patients report a preference for DTE over LT4, often due to better relief from symptoms such as fatigue, mental fog, and difficulties with weight management, which are not as effectively addressed by LT4 alone[32]. Additionally, genetic variations that impair the conversion of T4 to the more active T3 may make DTE, which directly supplies both hormones, particularly beneficial for these individuals [7]. Furthermore, some studies suggest that DTE may promote weight loss and improve metabolic parameters, possibly due to the direct metabolic effects of T3 [32]. While DTE may offer advantages for certain subgroups of patients, especially those who do not achieve optimal results with LT4, it should be used cautiously, tailored to individual needs, and supported by more definitive, large-scale clinical trials to fully understand its long-term efficacy compared to LT4.

Our metanalysis does not suggest any clinical benefits of DTE or combined T4 and T3 supplementation over T4 therapy alone in the broader population.

These results highlight the complexity of thyroid hormone replacement therapy and the need for individualized treatment approaches. While combined T4 + T3 therapy offers distinct biochemical changes, these do not translate into significant clinical differences in lipid profiles, heart rate, or most quality-of-life measures. However, the observed improvement in mental well-being with combined therapy could be a potential area for further exploration.

There are several strengths alongside notable weaknesses. Among its strengths is the study’s comprehensive literature search across multiple databases using a Boolean approach and specific keywords, which ensures a thorough collection of relevant studies. This is further enhanced by the exclusive focus on randomized controlled trials (RCTs), adhering to the highest standards of scientific evidence and significantly bolstering the reliability and validity of the findings. The rigorous data extraction process, conducted independently by multiple investigators and utilizing the Cochrane risk-of-bias tool (RoB 2), adds to the study’s thoroughness and accuracy, minimizing potential biases in the included clinical trials. The detailed statistical analysis using Review Manager (RevMan) software, mean differences, standardized mean differences, and forest plots provides a robust and clear statistical framework, enhancing the study’s credibility.

However, the study’s methodology is not without weaknesses. One significant issue is the high heterogeneity observed in some outcomes, suggesting substantial variability across studies, which could stem from differences in study populations, interventions, or methodologies. This raises questions about the generalizability of the findings. Furthermore, by excluding studies not published in English, the study potentially overlooks significant research, introducing language bias and raising concerns about publication bias, particularly regarding studies with negative or inconclusive results. These strengths and weaknesses collectively highlight the study’s comprehensive yet somewhat restricted approach to evaluating thyroid hormone therapies.

## Conclusion

Our meta-analysis provides a comprehensive evaluation of thyroid hormone replacement therapies in hypothyroidism, revealing that combined T4 + T3 therapy and desiccated thyroid extract (DTE) offer distinct biochemical profiles compared to T4 monotherapy yet do not significantly impact a range of clinical outcomes. Notably, combined therapy does not alter TSH levels but significantly affects total T4 and T3 levels, suggesting specific biochemical changes without corresponding clinical benefits regarding lipid profile, cardiovascular risk markers, and quality of life measures. However, a significant improvement in mental health and well-being with combined therapy emerges as a potential benefit, highlighting the need for personalized treatment strategies. The observed high heterogeneity across studies underscores the complexity of thyroid hormone therapy and the importance of individual patient factors in treatment decisions, paving the way for more tailored and nuanced approaches to managing hypothyroidism.

### Supplementary Information

Supplementary Material 1.

## Data Availability

All data avilable online.

## Abbreviations

BDI: Beck Depression Inventory
DTE: Desiccated thyroid extract
GHQ-28: General Health Questionnaire 28
GHQ-12: 12-Point quality of life general health questionnaire
LT4: Levothyroxine
LT3: Liothyronine
L4: Levothyroxine
MOOSE: Meta-analyses and Systematic Reviews of Observational Studies
T3: Triiodothyronine
T4: Thyroxine
RoB.2: Revised Cochrane risk-of-bias tool
SCL-90: Symptoms Checklist-90
SHBG: Sex hormone binding globulin
TG: Triglyceride
TSQ-36: 36-Point thyroid symptom questionnaire
TSH: Thyroid stimulating hormone or thyrotropin
TSQ-36: Post-treatment scores on the 36-point thyroid symptom questionnaire
VMS-IV: Wechsler memory scale-version IV
GHQ-28: General Health Questionnaire (GHQ)-28
