# Supplementary material for: Evaluating the effectiveness of combined T4 and T3 therapy or desiccated thyroid versus T4 monotherapy in hypothyroidism: a systematic review and meta-analysis
Source: BMC Endocr Disord. 2024 Jun 14;24:90. doi: 10.1186/s12902-024-01612-6 (PMC11177353; doi:10.1186/s12902-024-01612-6)
Supplement: Supplementary file 1 — Supplementary Material 1. [file 12902_2024_1612_MOESM1_ESM.docx]

Supplement Table 1: Search strategy

("Armour Thyroid" OR "Thyroid extract" OR "Natural desiccated thyroid" OR "Nature-Throid" OR "desiccated thyroid" OR "np thyroid" OR "Synthroid" OR "levothyroxine" OR "Liothyronine" OR "Cytomel" OR "Thyroid USP" OR "Unithroid" OR ("Natural desiccated thyroid" OR "Nature-Throid" OR "Armour Thyroid") AND ("Synthroid" OR "levothyroxine" OR "Liothyronine" OR "Cytomel" OR "Thyroid USP" OR "Unithroid") OR ("desiccated thyroid" OR "np thyroid") AND ("Synthroid" OR "levothyroxine" OR "Liothyronine" OR "Cytomel" OR "Thyroid USP" OR "Unithroid").

**Time: 11/23/2023**

| Database | Search terms | Search filed | Search results |
| --- | --- | --- | --- |
| Medline/  Pubmed | *("Armour Thyroid" OR "Thyroid extract" OR "Natural desiccated thyroid" OR "Nature-Throid" OR "desiccated thyroid" OR "np thyroid" OR "Synthroid" OR "levothyroxine" OR "Liothyronine" OR "Cytomel" OR "Thyroid USP" OR "Unithroid" OR ("Natural desiccated thyroid" OR "Nature-Throid" OR "Armour Thyroid") AND ("Synthroid" OR "levothyroxine" OR "Liothyronine" OR "Cytomel" OR "Thyroid USP" OR "Unithroid") OR ("desiccated thyroid" OR "np thyroid") AND ("Synthroid" OR "levothyroxine" OR "Liothyronine" OR "Cytomel" OR "Thyroid USP" OR "Unithroid")* | All filed | 903 |
| Web of Science | ("Armour Thyroid" OR "Thyroid extract" OR "Natural desiccated thyroid" OR "Nature-Throid" OR "desiccated thyroid" OR "np thyroid" OR "Synthroid" OR "levothyroxine" OR "Liothyronine" OR "Cytomel" OR "Thyroid USP" OR "Unithroid" OR ("Natural desiccated thyroid" OR "Nature-Throid" OR "Armour Thyroid") AND ("Synthroid" OR "levothyroxine" OR "Liothyronine" OR "Cytomel" OR "Thyroid USP" OR "Unithroid") OR ("desiccated thyroid" OR "np thyroid") AND ("Synthroid" OR "levothyroxine" OR "Liothyronine" OR "Cytomel" OR "Thyroid USP" OR "Unithroid") | All filed | 855 |
| Embase | ("Armour Thyroid" OR "Thyroid extract" OR "Natural desiccated thyroid" OR "Nature-Throid" OR "desiccated thyroid" OR "np thyroid" OR "Synthroid" OR "levothyroxine" OR "Liothyronine" OR "Cytomel" OR "Thyroid USP" OR "Unithroid" OR ("Natural desiccated thyroid" OR "Nature-Throid" OR "Armour Thyroid") AND ("Synthroid" OR "levothyroxine" OR "Liothyronine" OR "Cytomel" OR "Thyroid USP" OR "Unithroid") OR ("desiccated thyroid" OR "np thyroid") AND ("Synthroid" OR "levothyroxine" OR "Liothyronine" OR "Cytomel" OR "Thyroid USP" OR "Unithroid") | All filed | 4639 |

Supplement Table 2: Summary of included studies

| Study ID | Study group: intervention / Control | Study design | country | Center | Recruitment | Number of patients | Dose | Follow up | primary outcome | secondary outcome |
| --- | --- | --- | --- | --- | --- | --- | --- | --- | --- | --- |
| Shakir 2021 [9] | Combined T4/T3 | RCT cross-over | USA | Walter Reed National Military Medical Center | 2015-05 to 2018-05 | 75 | 83.6 ± 19.7 / 8.78 ± 1.74 Vs. 84.1 ± 19.6 / 8.82 ± 1.75 mcg/d | 22 weeks | TSQ-36, GHQ-12, VMS-IV, and BDI | treatment preference, biochemical and metabolic parameters, etiology of hypothyroidism, and Thr92Ala-DIO2 gene polymorphism |
|  | T4 |  |  |  |  | 75 | 115 ± 25 vs 115 ± 25 mcg/d |  |  |  |
| Fadeyev 2010 [15] | Combined T4/T3 | RCT | Russia | Moscow Medical Academy | - | 16 | L-T4 was reduced by 25 μg and replaced by 12.5 μg of L-T3 | 6 months | - | - |
|  | T4 |  |  |  |  | 20 | 1.6 μg per kg |  |  |  |
| Nygaard 2009 [16] | Combined T4/T3 | RCT cross-over | Denmark | Outpatient clinics at Herlev Hospital, Esbjerg Hospital, & Fredreriksberg Hospital | - | 59 | 50 µg T4 + 20 µg T3 | 24 weeks | Serum levels of TSH | Changes in thyroid function, weight, bioimpedance, waist-to-hip ratio, and quality of life. Depression scale, Side effects. |
|  | T4 |  |  |  |  | 59 | 50 µg T4 |  |  |  |
| Valizadeh 2009 [22] | Combined T4/T3 | RCT | Iran | Outpatient Clinic of Vali-Asr Hospital, Academic Medical Center | Aug 2006 to Dec 2007 | 30 | - | - | - | - |
|  | T4 |  |  |  |  | 30 | - |  |  |  |
| Regalbuto 2007 [33] | Combined T4/T3 | RCT | Italy | University of Catania | - | 20 | 19.5/5.7 μg | 6 months | Mean serum AST, ALT, SHBG, osteocalcin values, the clinical score, the systolic and diastolic performance, and the neurological and neuropsychological evaluations. | - |
|  |  |  |  |  |  |  | or 74.2/1.4 μg |  |  |  |
|  | T4 |  |  |  |  | 20 | 60 μg /day |  |  |  |
| Appelhof 2005 [34] | Combined T4/T3 10:1 | RCT | Netherlands | Academic Medical Centre, University of Amsterdam | Oct 2001 to Dec 2003 | 46 | 75 μg LT4 (100 –25 μg) and 15 μg of LT3 | 6 months | Subjective preference of study medication after 15 wk | scores on questionnaires on mood, fatigue, psychological symptoms, and a substantial set of neurocognitive tests. |
|  | Combined T4/T3 5:1 |  |  |  |  | 47 |  |  |  |  |
|  | T4 |  |  |  |  | 48 | 75 μg LT4 (100 –25 μg) and 7.5 μg of LT3 |  |  |  |
| Fadeyev 2005 [14] | Combined T4/T3 | RCT | Russia | Moscow Medical Academy | - | 16 | 50-100 μg L-T4&12.5 μg L-T3 | 6 months | FT4 levels & FT3 levels | - |
|  | T4 |  |  |  |  | 42 | 50-125 μg L-T4 |  |  |  |
| Morreale 2005 [13] | Combined T4/T3 | RCT cross-over | USA |  | Oct 2000 to Jan 2003 | 14 | 75 μg L-T4&5 μg L-T3 | 8 weeks | serum thyroid hormone levels results of quality-of-life and psychometric tests, and patients' preference | Multiple biological thyroid hormone endpoints |
|  | T4 |  |  |  |  | 14 | 100 μg/day L-T4. |  |  |  |
| Rodriguez 2005 [17] | Combined T4/T3 | RCT cross-over | USA | University of Texas Health Science Center at Houston | Jan 2002 to July 2003 | 30 | L-T4 - 50 mcg + 10 mcg L-T3 | 12 weeks | Fatigue (PFS scale) | Symptoms of depression, certain symptoms of hypothyroidism, working memory, serum thyroid hormone profile |
|  | T4 |  |  |  |  | 30 | normal dose of L-T4 |  |  |  |
| Siegmund 2004 [19] | Combined T4/T3 | RCT crossover | Germany | Outpatient clinic at Department of Endocrinology, University of Greifswald | - | 23 | T4/T3 (5% of patient’s T4 dose replaced by T3) | 6 months | Mood states & cognitive functioning (BDI, STAI-GX, SCL-90, EWL 60 S, Bf-S, FAW, digit span test, digit symbol test, visual scanning test d2) | - |
|  | T4 |  |  |  |  | 23 | T4 (100 -175 μg) |  |  |  |
| Clyde 2003 [12] | Combined T4/T3 | RCT | USA | National Naval Medical Center in Bethesda | May 2000 to Feb 2002 | 22 | L-T4 - 50 μg once daily + 7.5 μg L-T3 twice daily | 4 months | Standardized tests of neurocognitive function, HRQL | Weight, HR, bp, thyroid function (TSH, free T4, total T3), SHBG, cholesterol, triglycerides |
|  | T4 |  |  |  |  | 22 | daily levothyroxine 50 μg and began taking 25 μg levothyroxine |  |  |  |
| Sawka 2003 [18] | Combined T4/T3 | RCT | USA | Hamilton Health Sciences and the University of Medicine and Dentistry of New Jersey |  | 20 |  | - | - | - |
|  | T4 |  |  |  |  | 20 |  |  |  |  |
| Walsh 2003 [20] | Combined T4/T3 | RCT cross-over | Australia | Sir Charles Gairdner Hospital and Western Australian Center for Pathology and Medical Research | April 2000 to Nov 2002 | 110 | reduced daily T4 by 50 g and took study medication (either 50 g T4 or 10 g liothyronine) in addition to their reduced T4 dose | 4 weeks | to evaluate symptoms of hypothyroidism, quality of life, cognitive function, and subjective satisfaction with T4 therapy. | - |
|  | T4 |  |  |  |  | 110 |  |  |  |  |
| Bunevicius 2002 [35] | Combined T4/T3 | RCT | USA | Institute of Endocrinology of Kaunas Medical University |  | 10 | substitution of 10 μg of T3 for 50 μg of T4 | 5 Weeks | - | - |
|  | T4 |  |  |  |  | 10 |  |  |  |  |
| Bunevicius 1999 [21] | Combined T4/T3 | RCT cross-over | USA | Institute of Endocrinology of Kaunas Medical University |  | 16 | 50 μg of thyroxine was replaced by 12.5 μg of triiodothyronine | 5 Weeks | mood state and neuropsychological function | - |
|  | T4 |  |  |  |  | 17 |  |  |  |  |
| Shakir 2021 [9] | desiccated thyroid | RCT cross-over | USA | Walter Reed National Military Medical Center | 2015-05 to 2018-05 | 75 | 76.7 ± 16.3 / 77.3 ± 16.8 | 22 weeks | TSQ-36, GHQ-12, VMS-IV, and BDI | treatment preference, biochemical and metabolic parameters, etiology of hypothyroidism, and Thr92Ala-DIO2 gene polymorphism |
|  | T4 |  |  |  |  | 75 | 115 ± 25 vs 115 ± 25 mcg/d |  |  |  |
| Hoang 2013 [32] | desiccated thyroid | RCT cross-over | USA | Walter Reed National Military Medical Center, Bethesda |  | 70 | 38 μg L-T4 and 9 μg liothyronine | 6 months | TSQ, the GHQ-12, the WMS-IV, and the BDI | - |
|  | T4 |  |  |  |  | 70 |  |  |  |  |

Supplement Table 3: Baseline characteristics

| Study ID | Study group: intervention / Control | Age, y | Male | Autoimmune hypothyroidism | Post-surgical hypothyroidism | Total cholesterol | LDL cholesterol | HDL cholesterol | TG |
| --- | --- | --- | --- | --- | --- | --- | --- | --- | --- |
| Shakir 2021 [9] | Combined T4/T3 | 50 (range 29-65) | 17 (22.7%) | 46 (61.3%) | 16 (21.3%) | 199 ± 39.8 | 128 ± 36.0 | 61.0 ± 18.6 | 106 ± 60.7 |
|  | T4 |  |  |  |  |  |  |  |  |
| Fadeyev 2010 [15] | Combined T4/T3 | 38.7 ± 8.9 | - | - | - | 6.2 ± 1.3 | 4.1 ± 0.7 | - | - |
|  | T4 | 41±8.9 | - | - | - | 5.6 ± 1.7 | 4 ± 1.6 | - | - |
| Nygaard 2009 [16] | Combined T4/T3 | 46.5 ±13.1 | 4 (6.78%) | 59 (100%) | - | - | - | - | - |
|  | T4 | 47.6 ±12.3 |  |  | - | - | - | - | - |
| Valizadeh 2009 [22] | Combined T4/T3 | 39.2 ±11.2 | 6 (20%) | 22 (73.3%) | 1 (3.3%) | 191 ±33 | 114 ±24 | 43 ±7 | 166 ± 64 |
|  | T4 | 38.8 (11.7) | 6 (20%) | 24 (80.0%) | 1 (3.3%) | 198 ±36 | 122 ±29 | 43 ±5 | 163 ±72 |
| Regalbuto 2007 [33] | Combined T4/T3 | 46.4 ± 8.6 | 3 (15%) | - | 20 (100%) | - | - | - | - |
|  | T4 |  |  | - |  | - | - | - | - |
| Appelhof 2005 [34] | LT4/LT3 10:1 | 46.8 ± 9.8 | 8 (17%) | - | - | 201 ± 39 | - | - | - |
|  | LT4/LT3 5:1 | 49.8 ± 9.4 | 6 (13%) | - | - | 215 ± 46 | - | - | - |
|  | T4 | 48.5 ± 9.4 | 7 (15%) | - | - | 206 ± 33 | - | - | - |
| Fadeyev 2005 [14] | Combined T4/T3 | - | 0 (0%) | - | - | - | - | - | - |
|  | T4 | - | - | - | - | - | - | - | - |
| Morreale 2005 [13] | Combined T4/T3 | 48 ± 11 | 8 (29%) | - | - | - | - | - | - |
|  | T4 |  |  | - | - | - | - | - | - |
| Rodriguez 2005 [17] | Combined T4/T3 | 47.5 ±12.9 | 5 (17%) | 23 (73%) | - | - | - | - | - |
|  | T4 |  |  |  | - | - | - | - | - |
| Siegmund 2004 [19] | Combined T4/T3 | 23-69 | 3 (13.04%) | 2 (8.70%) | - | 5.93 ± 1.36 | 3.88 ± 1.24 | 1.36 ± 0.35 | 1.50 ± 0.68 |
|  | T4 |  |  |  | - |  |  |  |  |
| Clyde 2003 [12] | Combined T4/T3 | 43.1 ± 11.3 | 3 (14%) | 13 (59.01%) | 0 (0%) | 206 ±51 | 121 ± 47 | 61 ±18 | 120 ±55 |
|  | T4 | 45.2 ± 9.7 | 5 (23%) | 18 (81.8%) | 1 (4.55%) | 206 ± 37 | 121 ± 32 | 47 ±16 | 188 ±108 |
| SAWKA 2003 [18] | Combined T4/T3 | - | - | - | - | - | - | - | - |
|  | T4 | - | - | - | - | - | - | - | - |
| WALSH 2003 [20] | Combined T4/T3 | 51.7 ± 11.6 | 68 (61%) | 44 (90%) | 3 (6%) | - | - | - | - |
|  | T4 | 44.4 ±10.7 | 51 (46%) | 50 (82%) | 9 (15%) | - | - | - | - |
| Bunevicius 2002 [35] | Combined T4/T3 | - | - | - | - | - | - | - | - |
|  | T4 | - | - | - | - | - | - | - | - |
| Bunevicius 1999 [21] | Combined T4/T3 | 48±15 | 0 (0%) | 12 (75%) | - | - | - | - | - |
|  | T4 | 45±10 | 2(11%) | 4 (23.5%) | - | - | - | - | - |
| Shakir 2021 [9] | desiccated thyroid | 50 (range 29-65) | 17 (22.7%) | 46 (61.3%) | 16 (21.3%) | 199 ± 39.8 | 128 ± 36.0 | 61.0 ± 18.6 | 106 ± 60.7 |
|  | T4 |  |  |  |  |  |  |  |  |
| Hoang 2013 [32] | desiccated thyroid | 50.66 (range 23– 65) | 17 (24.29%) | 35 (50.0%) | 8 (11.43) | 194.77 ± 33.50 | 114.33 ± 29.53 | 60.70 ± 15.92 | 102.57 ± 59.88 |
|  | T4 |  |  |  |  |  |  |  |  |
| Study ID | **Study group: intervention / Control** | **Total T3** | **T3 resin uptake** | **T3 reverse** | **TSH** | **Total T4** | **Free T4** | **SHBG** | **HR** |
| Shakir 2021 [9] | Combined T4/T3 | 109 ± 27.3 | 28.0 ± 3.53 | 19.9 ± 6.81 | 1.77 ± 1.10 | 8.21 ±2.22 | 1.41 ± 0.37 | 71.0 ± 51.7 | - |
|  | T4 |  |  |  |  |  |  |  |  |
| Fadeyev 2010 [15] | Combined T4/T3 | - | - | - | 45.2 ± 49.1 | - | 8.1 ± 3.4 | - | 76 ± 5.2 |
|  | T4 | - | - | - | 42.3 ± 43.6 | - | 7.9 ± 4.3 | - | 78.3 ± 5.2 |
| Nygaard 2009 [16] | Combined T4/T3 | - | - | - | 1.286 (1.2333) | - | 124 (29) | - | - |
|  | T4 | - | - | - |  | - |  | - | - |
| Valizadeh 2009 [22] | Combined T4/T3 | 164 (14) | - | - | 2.5 (1.2) | 6.7 (1.1) | - | - | 79 (7) |
|  | T4 | 134 (27) | - | - | 2.1 (1.2) | 7.9 (1.7) | - | - | 78 (6) |
| Regalbuto 2007 [33] | Combined T4/T3 | - | - | - | - | - | - | - | - |
|  | T4 | - | - | - | - | - | - | - | - |
| Appelhof 2005 [33] | LT4/LT3 10:1 | 1.27 ± 1.29 | - | - | - | - | 1.15 ± 0.26 | 55 ± 37 | - |
|  | LT4/LT3 5:1 | 0.38 ± 0.79 | - | - | - | - | 1.18 ± 0.24 | 53 ± 35 | - |
|  | T4 | 1.02+-0.87 | - | - | - | - | 1.15 ± 0.18 | 55 ± 32 | - |
| Fadeyev 2005 [14] | Combined T4/T3 | - | - | - | - | - | - | - | - |
|  | T4 | - | - | - | - | - | - | - | - |
| Morreale 2005 [13] | Combined T4/T3 | - | - | - | - | - | - | - | - |
|  | T4 | - | - | - | - | - | - | - | - |
| Rodriguez 2005 [17] | Combined T4/T3 | 79.0 (18.0) | - | - | 1.9 (1.7) | 10.9 (2.0) | 3.2 (0.1) | - | - |
|  | T4 |  | - | - |  |  |  | - | - |
| Siegmund 2004 [19] | Combined T4/T3 | - | - | - | 1.72 (1.23) | - | 22.1 (3.37) | 4.71 (3.07) | 69.3 (12.5) |
|  | T4 | - | - | - |  | - |  |  |  |
| Clyde 2003 [12] | Combined T4/T3 | 89 (16) | - | - | 2.6 (2.0) | - | 1.3 (0.2) | 60 (32) | 73 (12) |
|  | T4 | 96 (18) | - | - | 2.2 (2.1) | - | 1.2 (0.2) | 56 (58) | 72 (11) |
| Sawka 2003 [18] | Combined T4/T3 | - | - | - | - | - | - | - | - |
|  | T4 | - | - | - | - | - | - | - | - |
| Walsh 2003 [20] | Combined T4/T3 | 3.3 (0.8) | - | - | - | 15.3 (2.3) | - | - | - |
|  | T4 | 3.5 (0.9) | - | - | - | 15.4 (2.2) | - | - | - |
| Bunevicius 2002 [35] | Combined T4/T3 | - | - | - | - | - | 20.7 ± 6.2 | - | - |
|  | T4 | - | - | - | - | - |  | - | - |
| Bunevicius 1999 [21] | Combined T4/T3 | - | - | - | - | - | 1.9±0.5 | - | - |
|  | T4 | - | - | - | - | - | 2.0±0.7 | - | - |
| Shakir 2021 [9] | desiccated thyroid | 109 ± 27.3 | 28.0 ± 3.53 | 19.9 ± 6.81 | 1.77 ± 1.10 | 8.21 ±2.22 | 1.41 ± 0.37 | 71.0 ± 51.7 | - |
|  | T4 |  |  |  |  |  |  |  |  |
| Hoang 2013 [32] | desiccated thyroid | 94.62 ± 25.16 | 31.81 ± 3.32 | 32.26 ± 12.85 | 1.69 ± 0.78 | 9.10 ± 2.11 | 1.34 ± 0.27 | 64.41 ± 45.98 | 73.36 ± 11.31 |
|  | T4 |  |  |  |  |  |  |  |  |


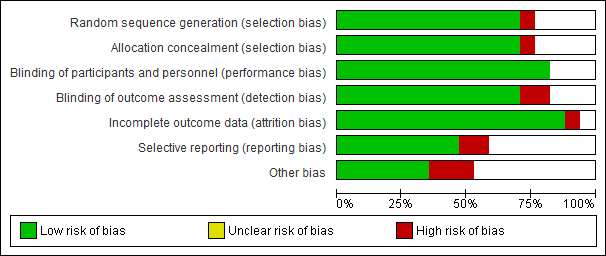


Supplementary Figure 1: Summary of bias risk in studies comparing the effects of combined T4+T3 therapy versus T4 monotherapy.


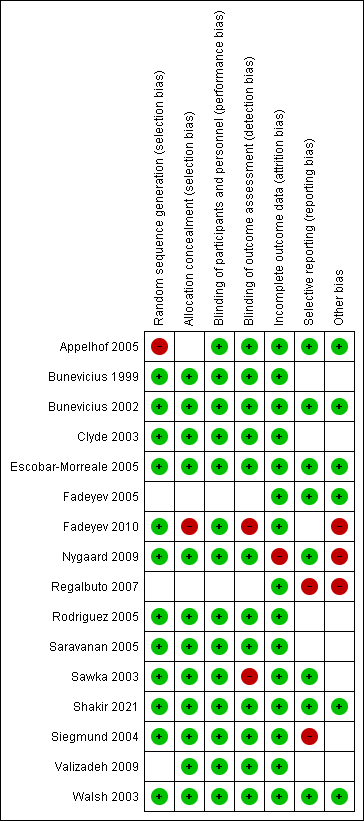


Supplementary Figure 2: Risk of Bias Assessment for Selected Studies of combined T4+T3 therapy versus T4 monotherapy.


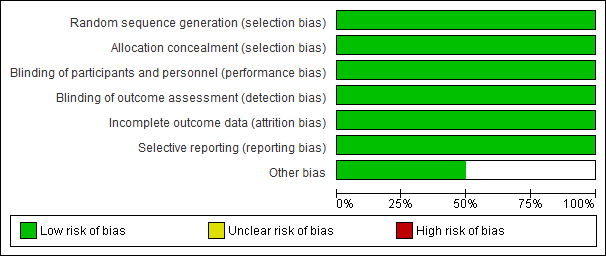


Supplementary Figure 3: Summary of bias risk in studies comparing the effects of combined DTE therapy versus T4 monotherapy.


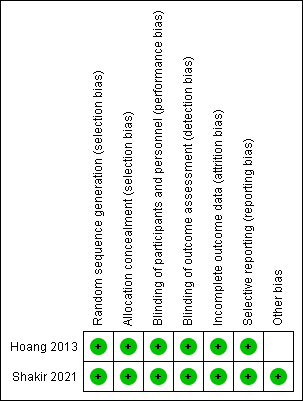


Supplementary Figure 4: Risk of Bias Assessment for Selected Studies of combined DTE therapy versus T4 monotherapy.
